# Supplementary material for: Open acid dissolution—Ammonia solution extraction—ICP OES rapid determination of 7 trace metal elements in soil
Source: PLoS One. 2023 Oct 10;18(10):e0292168. doi: 10.1371/journal.pone.0292168 (PMC10564144; doi:10.1371/journal.pone.0292168)
Supplement: Attachment — Submitted filename: Response to Reviewers.docx [file pone.0292168.s001.docx]

Dear Editor, Reviewer：

Thankyou very much for your suggestions and comments on my paper. Here I will address each of the scientific issues in my paper as requested by the reviewers and the answers have been highlighted.

Reviewer #1:

The manuscript entitled “Open acid dissolution - Ammonia solution extraction - ICP-OES rapid determination of 7 heavy metal elements in soil” report on a new method for to overcome the corrosion of hydrofluoric acid on the ICP-OES injection system in the acid dissolution system. It presents scientific relevance for the area of Chemistry, Biology, and others area.

The manuscript presents an interesting proposal, but from determining V, Cr, Co, Ni, Cu, Zn and Pb if digestion with HF is necessary? These analytes can be extracted with a mixture of strong acids (such as HNO3, HCl, H2SO4, etc…) and oxidizing agents! If the proposal were to determine silicon, the use of HF is justified!

The use of a mixed acid solution containing hydrofluoric acid (HF) for dissolution and subsequent measurement by ICP-OES or ICP-MS is the primary method for determining the values of almost all soil standard substances in China. If other methods, such as those used by the EPA in other countries, which involve extraction with nitric acid (HNO3) and hydrochloric acid (HCl) followed by measurement, are used, the measured values may not be consistent with the certified values of Chinese standard substances. Therefore, hydrofluoric acid was used as the dissolving agent in this study.

The language (English) are satisfactory (I suggest the final revision)! However, you need to change some details/information in the abstract, Introduction, Methods, results, discussion and conclusions.

1. Title: Adequate! To replace “ICP-OES” by “ICP OES”, and throughout the entire manuscript.

Modified

2. Abstract: Adequate, but I suggest rewrite and add information:

- To replace “μg/g” by “mL min-1” and “μg g-1”, and throughout the entire manuscript.

Modified

- What are the quantification limit values?

Due to an error by the author, the term "detection limits" in the original text actually refers to what the reviewer considered to be "quantification limits". This has now been corrected.

- I suggest highlighting the "innovative" proposal of the study.

The opening and closing statements of the Abstract, the last paragraph of the Introduction and the Conclusion all emphasise the novelty and beneficial effects of this study. The author considers this to be sufficient.

3. Introduction section: It is well written, but I suggest:

- The term “heavy metals” is not appropriate. The term “heavy metals” is the subject of many discussions. I suggest that the term be replaced, throughout the manuscript, according to [Science of the Total Environment 610–611 (2018) 419–420: “Heavy metal” - What to do now: To use or not to use?] and [Hazrat Ali & Ezzat Khan (2018) What are heavy metals? Long-standing controversy over the scientific use of the term ‘heavy metals’ – proposal of a comprehensive definition, Toxicological & Environmental Chemistry, 100:1, 6-19, DOI: 10.1080/02772248.2017.1413652]. Therefore, I strongly suggest removal of “heavy metals” from all text and replacement in the abstract and full text of submitted paper with words like “potentially toxic metal(s)/element(s)” or “trace metal(s)/element(s)”, according to the context, throughout the manuscript.

Heavy metal (s) has been changed to Trace metal (s)

- I suggest inserting more information and references about: To avoid damage to the ICP OES injection system from hydrofluoric acid; and, alkaline fusion methods.

A reference to the potential damage to the ICP OES injection system caused by hydrofluoric acid has been added.

For alkaline fusion methods, a reference is already included in the third paragraph of the Introduction, so no additional reference is needed here.

- I suggest highlighting the "innovative" proposal of the study, as well as the advantages / disadvantages, at the end of the introduction.

The shortcomings of this method have been added at the end of the Conclusion.

4. Material and Methods section:

- I suggest inserting more information about the mineral composition of the national standard substances.

The information has been added to the 'Main reagents and materials' section.

- In “Experimental method”: I request more information about the sample storage time until analysis, etc. How were the digestion conditions optimized? Did you follow any published article/pervious protocol? If yes, insert reference!

The samples used in this study were soil samples, which do not require special storage conditions compared to biological samples. Therefore, information such as storage time has not been emphasised in the text.

Optimisation of digestion conditions has been added to the text based on the following references.

- In “Experimental method”: The authors wrote: “The national standard substance GSS2, digested simultaneously with the sample, was used as the peak and the blank solution as the valley. The two-point standard working curve for each element was obtained by measuring the solutions under the selected instrument operating conditions. The test solution was then measured under the same conditions”. I suggest a better explanation for: "peak and the blank solution as the valley" and "The two-point standard working curve for each element...". Was the curve plotted with only 2 points? How were the precision tests carried out? How many concentration levels were used? The authors proposed the validation of an analytical method, robustness, etc. What concentration levels are used to assess accuracy? Ideally, 3 concentration levels (low, medium, and high) should be used to assess accuracy. Robustness? I suggest detailing the proposed method in more detail...

Typically, the purpose of a multi-point curve is to prevent the curve from bending, and this is commonly used in instruments such as atomic absorption spectrometers and atomic fluorescence spectrometers. However, one of the major advantages of ICP OES is its large linear range, and there is not much difference between a two-point curve and a multi-point curve. Of course, to avoid errors when measuring unknown samples using a two-point curve, it is necessary to measure quality control samples first for verification. This explanation has been added to the text.

- The authors proposed the validation of an analytical method, however there are no details about this step! What are the analytical validation parameters used? Has the proposed method been validated? If so, which protocol / guidelines (IUPAC, ICH, ETC.) did you follow? What are the validation parameters studied? Precision, accuracy, LOD, LOQ, robustness, etc. What concentration levels are used to assess accuracy? I suggest detailing the proposed method in more detail...

Quantitative limits and precision data are presented in the 'Method quantification limit and precision' and 'Accuracy of the method' sections of the 'Results and discussion', and the accuracy of the method is verified using soil standard materials. The measured values are in agreement with the certified values, and since the concentrations of the elements in the soil standard materials are similar to those in the actual samples, we have reason to believe that the accuracy of this method is reliable.

Reviewer #2

- In “Calibration curves” section: The authors wrote: “Therefore, in this study, the national standard substance GSS2 was used as the peak and the process blank as the valley to draw the calibration curves.” I suggest expanding the discussions with more details!

Corresponding basis has been added.

- In “Concentration of ammonia solution” section: How were the ammonia concentrations selected? Did you follow any published article/pervious protocol? If yes, insert reference!

The use of ammonia as a medium for the determination of elements such as bromine and iodine is supported by several references. However, the author has not found any specific references to the use of ammonia as a medium for the determination of metallic elements. As stated in the manuscript, the concentration of ammonia in the solution is determined by the pH value after its addition. The measurement can only be carried out under alkaline conditions, as fluoride ions can cause considerable damage to the injection system.

- In “Composition of the digestion acid” section: How were acid combinations defined? Univariate analysis? Multivariate? Did you follow any published article/pervious protocol? If yes, insert reference!

The author determined the ratio and dosage range of nitric acid and hydrofluoric acid based on existing literature and then made adjustments accordingly. Relevant references have been added to the manuscript.

- In “Method detection limit and precision” section: And the quantification limits?

Added.

I suggest expanding the discussions, comparing the data with the scientific literature. I suggest, at the end of the section, to write a paragraph summarizing the findings and their impacts on the research proposal.

The significance and innovative aspects of this study were mentioned several times in the abstract, introduction and conclusion.

6. Material and Methods section:

- Conclusion section: The conclusion is written in topics!!!??? I suggest highlighting the "innovative" proposal of the study, as well as the advantages/ disadvantages/limitations.

The limitations and drawbacks of this method have been added to the conclusion.

* Tables and Figures: Few tables and fihures.

To save space, many data such as precision and quantification limits are presented in the main text, and the use of tables and figures has been deliberately minimised.

* References: Please, check if the references are in accordance with the journal's rules.

Checked
