# Supplementary material for: Open acid dissolution—Ammonia solution extraction—ICP OES rapid determination of 7 trace metal elements in soil
Source: PLoS One. 2023 Oct 10;18(10):e0292168. doi: 10.1371/journal.pone.0292168 (PMC10564144; doi:10.1371/journal.pone.0292168)
Supplement: Attachment — Submitted filename: Response to Reviewers.docx [file pone.0292168.s002.docx]

Dear Editor, Reviewer：

Thankyou very much for your suggestions and comments on my paper. Here I will address each of the scientific issues in my paper as requested by the reviewers and the answers have been highlighted.

Reviewer #1:

The authors improved the manuscript based on the suggestions.

1.I suggest expanding the "discussions", comparing the results with the scientific literature. In the discussion only 3 references are cited (30-32)! In "Response to Reviewers" authors wrote "The use of a mixed acid solution containing hydrofluoric acid (HF) for dissolution and subsequent measurement by ICP-OES or ICP-MS is the primary method for determining the values of almost all soil standard substances in China. If other methods, such as those used by the EPA in other countries, which involves extraction with nitric acid (HNO3) and hydrochloric acid (HCl) followed by measurement, are used, the measured values may not be consistent with the certified values of Chinese standard substances. Therefore, hydrofluoric acid was used as the dissolving agent in this study". Therefore, it is necessary to compare the manuscript data with these studies.

GSS1a~GSS8a are relatively new national standard substances in China, only determined in 2020. There is little relevant reference, and no one will use other countries' methods to determine China's national standard substances. Therefore, this article can only compare the measured values of this method with those of the soil recommendation method in China to further prove the accuracy of this method.

2.Insert the values of standard deviations (+/- sd) in Table 2!

Modified.
